# Oxygen depletion recorded in upper waters of the glacial Southern Ocean

**DOI:** 10.1038/ncomms11146

**Published:** 2016-03-31

**Authors:** Zunli Lu, Babette A. A. Hoogakker, Claus-Dieter Hillenbrand, Xiaoli Zhou, Ellen Thomas, Kristina M. Gutchess, Wanyi Lu, Luke Jones, Rosalind E. M. Rickaby

**Affiliations:** 1 Department of Earth Sciences, Syracuse University, Syracuse, New York 13244, USA; 2 Department of Earth Sciences, University of Oxford, Oxford OX1 3AN, UK; 3 British Antarctic Survey, Cambridge CB3 0ET, UK; 4 Department of Geology and Geophysics, Yale University, New Haven, Connecticut, USA

## Abstract

Oxygen depletion in the upper ocean is commonly associated with poor ventilation and storage of respired carbon, potentially linked to atmospheric CO_2_ levels. Iodine to calcium ratios (I/Ca) in recent planktonic foraminifera suggest that values less than ∼2.5 μmol mol^−1^ indicate the presence of O_2_-depleted water. Here we apply this proxy to estimate past dissolved oxygen concentrations in the near surface waters of the currently well-oxygenated Southern Ocean, which played a critical role in carbon sequestration during glacial times. A down-core planktonic I/Ca record from south of the Antarctic Polar Front (APF) suggests that minimum O_2_ concentrations in the upper ocean fell below 70 μmol kg^−1^ during the last two glacial periods, indicating persistent glacial O_2_ depletion at the heart of the carbon engine of the Earth's climate system. These new estimates of past ocean oxygenation variability may assist in resolving mechanisms responsible for the much-debated ice-age atmospheric CO_2_ decline.

## Abstract (web summary)

Oxygen depletion in the ocean interior may be closely linked to atmospheric CO_2_ level, yet robust proxies are scarce. Here, using foraminiferal I/Ca as a proxy for oxygenation changes, the authors show that oxygen depletion in the upper waters of the high latitude Southern Ocean occurred during glacials.

## Graphical abstract

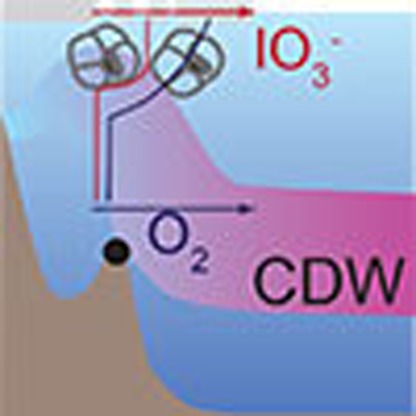

The Southern Ocean is widely considered to be critical to global nutrient and carbon cycling, including over glacial–interglacial time scales^1^. As an area of incomplete nutrient utilization, it is a major source of CO_2_ to the atmosphere today^2^. At present, old (CO_2_− and nutrient-rich and relatively O_2_−depleted) deep waters upwell along most of the Antarctic continental margin^3^,^4^ (Fig. 1), release CO_2_ into, and recharge O_2_ from surface waters before they down-well in distinct areas, such as the Weddell and Ross seas, to form Antarctic Bottom Water (AABW). In the glacial Southern Ocean, strengthening of the biological pump due to enhanced iron supply^5^,^6^, increased stratification^7^, and expanded sea–ice cover^8^, were among the dominant players in reducing atmospheric CO_2_ by ∼90 p.p.m.V. Each of these mechanisms could counterbalance the increased O_2_ solubility due to lower glacial temperatures, leading to a reduction in the O_2_ concentration of the seawater. Since the Southern Ocean is thought to have reduced its CO_2_ leakage during glacial periods^1^, it provides an ideal location to search for evidence of deoxygenation linked to CO_2_ sequestration in the upper ocean.

During the last glacial period, deep waters surrounding Antarctica were less ventilated, and older than today (relative to the atmosphere)^9^. A recent quantitative O_2_ proxy study based on benthic foraminiferal δ^13^C indicates that decreased ventilation linked to a reorganization of glacial ocean circulation and a strengthened global biological pump significantly enhanced the ocean storage of respired carbon in the deep North Atlantic^10^. Early box-models hypothesized very low-oxygen levels in the high latitude Southern Ocean^11^,^12^. Proxies did not paint a clear picture for bottom-water O_2_ concentrations in the glacial Southern Ocean^13^. Only a few studies on marine sediment cores south of the APF have found evidence for substantially lowered bottom water O_2_ concentrations. There, authigenic uranium concentrations were elevated in sediments deposited during glacial Marine Isotope Stages (MIS) 2 and 6 (refs 14, 15). By contrast, another study highlighted a transient stagnation event during the early stage of the last interglacial (MIS 5e)^16^.

Bottom water or porewater redox proxies cannot capture upper ocean O_2_ levels far from the continental shelf, so there is scant constraint on upper ocean oxygenation conditions in vast tracts of the open ocean^13^. A novel proxy, the I/Ca composition of marine carbonates, especially planktonic and benthic foraminiferal tests, has demonstrated its potential to reconstruct paleo-oxygenation levels in both the upper ocean^17^,^18^,^19^,^20^ and bottom waters^21^, respectively. The thermodynamically stable forms of iodine in seawater are iodate (IO_3_^−^) and iodide (I^−^)^22^. The total concentrations of IO_3_^−^ and I^−^ are relatively uniform in the world ocean at around 0.45 μmol l^−1^ due to the residence time of ∼300 kyr (ref. 23), supported by a more recent compilation of iodine concentrations in global rivers^24^. Therefore, the total iodine concentration in the global ocean likely remained invariant over the duration of a glacial termination (∼6 kyr).

Iodate is taken up by marine organisms as a micronutrient in surface waters^25^, but its concentration does not increase during the aging of deep waters^26^,^27^, in contrast to those of the major nutrients nitrate and phosphate, probably due to the low I/C_org_ ratio of plankton^25^. Iodine speciation is strongly redox sensitive. IO_3_^−^ is completely converted to I^−^ when oxygen is depleted^28^. Because IO_3_^−^ is the only chemical form of iodine that is incorporated into the structure of carbonate^17^, calcareous tests precipitated closer to an oxygen minimum zone (OMZ) will record lower I/Ca and vice versa. An OMZ is defined by O_2_<20 μmol kg^−1^ in the Pacific Ocean and O_2_<50 μmol kg^−1^ in the Atlantic Ocean^29^.

In this paper, we use recent planktonic foraminifera and modern water column data to establish typical I/Ca values for the presence of an OMZ or O_2_-depleted water. On the basis of this proxy development, the down-core record of planktonic foraminifera I/Ca obtained at site TC493/PS2547 indicates the persistent presence of oxygen-depletion in the upper waters of high latitude Southern Ocean during the last two glacial periods.

## Results

### Site selection

**Figure 1:**
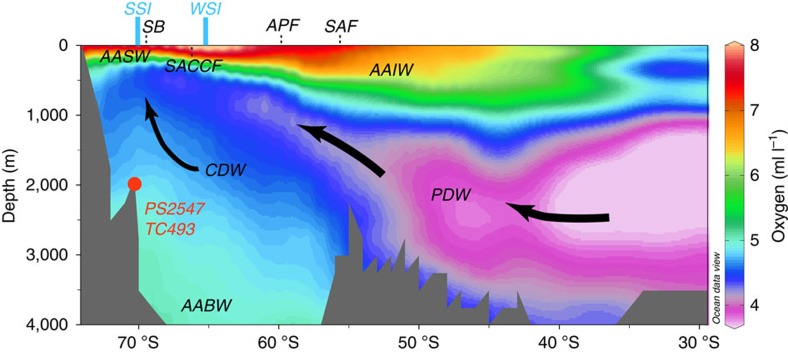
Hydrographic section of Southern Ocean in the Pacific sector. Dissolved oxygen concentrations showing major water masses^50^ and boundaries, average modern summer (SSI) and winter (WSI) sea–ice extent^62^, and core site PS2547/TC493. The locations of the Antarctic Circumpolar Current (ACC) fronts are marked as SB, Southern Boundary of the ACC; SACCF, Southern ACC Front; APF, Antarctic Polar Front; SAF, Sub-Antarctic Front. AABW, Antarctic Bottom Water; AAIW, Antarctic Intermediate Water; AASW, Antarctic Surface Water; CDW, Circumpolar Deep Water; PDW, Pacific Deep Water. This graph is generated in Ocean Data View, using the Southern Ocean Atlas data set^63^.

We measured I/Ca values on eleven planktonic foraminiferal species in modern to Holocene samples, and in one sample from a previous interglacial (Supplementary Table 1 and Supplementary Figs 1 and 2). We chose sites from well-oxygenated areas (for example, the North and sub-Antarctic South Atlantic), and sites located beneath OMZs, including Ocean Drilling Program (ODP) Sites 658, 709, 720 (Site 720: last interglacial samples), 849 and 1242. First, we use these data to further establish the foundations of the I/Ca proxy. Subsequently, we focus on an I/Ca down-core record on *Neogloboquadrina pachyderma* sinistral deposited during the last two glacial cycles in two sediment cores (PS2547 and TC493) recovered from the same location (71°09′ S, 119°55′ W, water depth 2,096 m) on a seamount in the Amundsen Sea (Fig. 1)^30^. The excellent carbonate preservation at this site^30^ provides a unique window to reconstruct past upper ocean conditions south of the APF. Site TC493/PS2547 is currently bathed by Circumpolar Deep Water (CDW), which is overlain by a layer of Antarctic Surface Water (AASW), or Winter Water^31^,^32^,^33^, and is located on the edge of the average modern summer sea–ice limit^34^ (Fig. 1). During the Last Glacial Maximum (LGM), the sea–ice boundaries within the Southern Ocean shifted significantly northwards^35^,^36^. Thus, it is highly likely that site TC493/PS2547 was located within the permanent sea–ice zone during past glacial periods^34^.

### Age model and glacial polynyas

The sediments of core TC493/PS2547 consist mainly of foraminiferal ooze and sandy mud, with *N. pachyderma* (s) tests forming the primary carbonate component^30^. The age model of the record is based on magnetostratigraphy combined with benthic foraminiferal (*Cibicides* cf. *wuellerstorfi*) oxygen isotope (δ^18^O) stratigraphy^30^, tuned to the global benthic δ^18^O stack^37^. Continuous deposition of foraminifera^30^ indicates at least episodic opening of polynyas during glacial periods^34^, because of its seamount location^38^,^39^. This scenario is consistent with the occurrence of the benthic foraminifera species *Epistominella exigua*, which is adapted to highly episodic phytodetritus supply^40^.

### I/Ca in foraminifera

**Figure 2:**
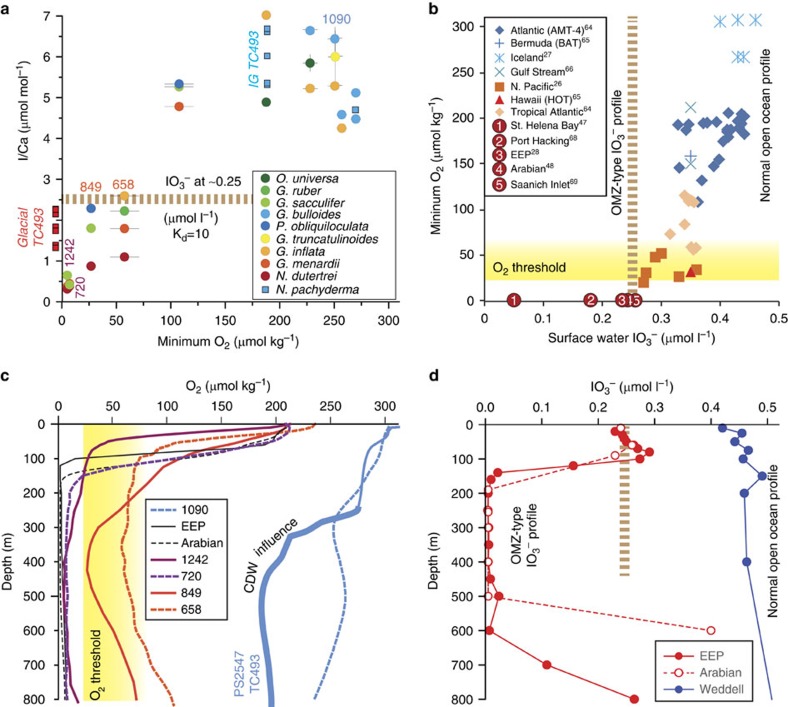
I/Ca and modern OMZs. (**a**) Modern and late Holocene I/Ca in planktonic foraminiferal tests versus minimum O_2_ concentrations in the upper 500 m of the water column (Note: I/Ca at Site 720 is from a MIS 5 sample). Error bars for *y* axis indicate the s.d. (1 s.d.) of triplicate measurements. Blue squares show down-core interglacial (IG) I/Ca data on *N. pachyderma* (s) from site TC493/PS2547 plotted against minimum O_2_ concentrations in the modern water column, indicating well-oxygenated conditions. I/Ca for glacial *N. pachyderma* (s) tests are marked as red squares, indicating O_2_ depletion. (**b**) Compilation of modern ocean surface water IO_3_^−^ concentrations compared with minimum O_2_ concentrations^26^,^27^,^28^,^47^,^48^,^64^,^65^,^66^,^67^,^68^,^69^. Brown dashed line indicates the surface water IO_3_^−^ concentration of ∼0.25 μmol l^−1^ as a threshold value for differentiating OMZ-type and normal open ocean type of IO_3_^−^ depth profiles. (**c**) O_2_ depth profiles. Yellow shading marks 20–70 μmol kg^−1^ O_2_ concentration as the threshold for complete iodate reduction. (**d**) IO_3_^−^ depth profiles at OMZ sites from the Eastern Equatorial Pacific (EEP)^28^ and the Arabian Sea (station N8)^48^ and at a well-oxygenated high-latitude site near the Weddell Sea (station PS71/179–1)^54^.

**Figure 3:**
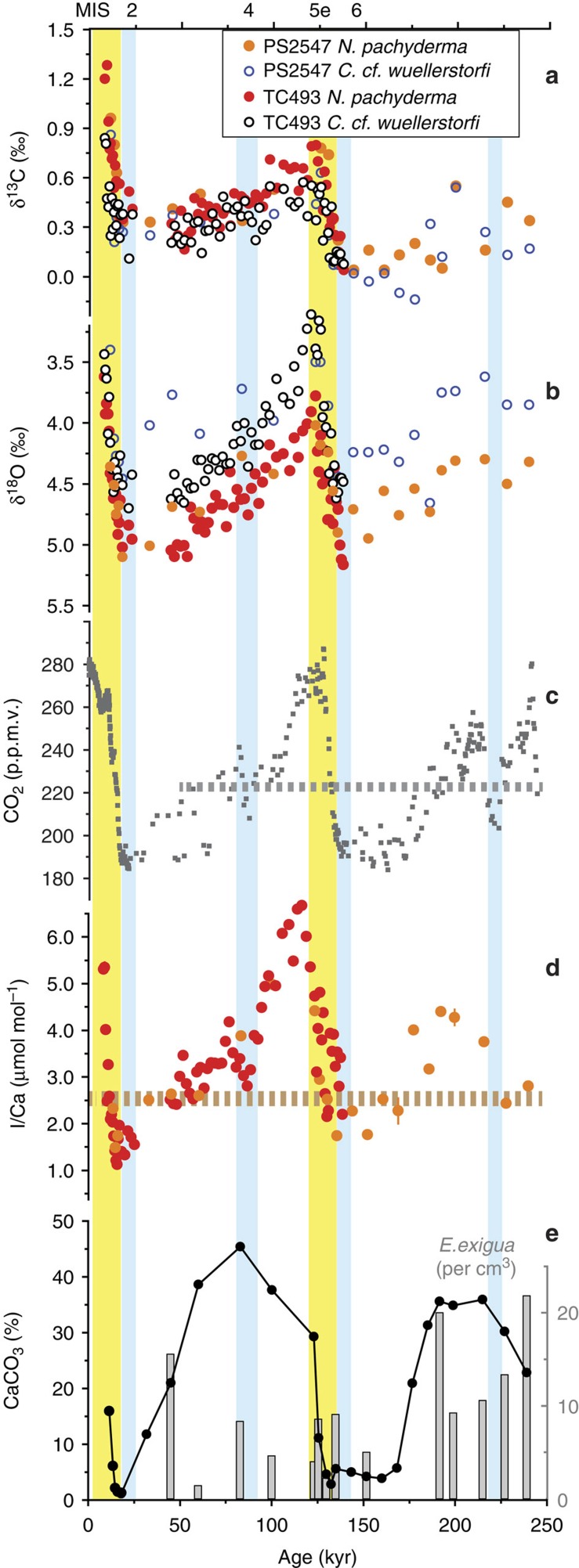
Down-core records of studied sites. (**a**,**b**) Stable carbon and oxygen isotopes measured on benthic (*C.* cf. *wuellerstorfi*) and planktonic (*N. pachyderma*) foraminiferal tests^30^. It is well documented that δ^13^C of *N. pachyderma* (s) is offset by −1.0‰ south of the APF^49^ and the values plotted here are uncorrected. (**c**) Atmospheric pCO_2_ record at EPICA Dome C is plotted for comparison, with dashed line indicating the long-term mean value of CO_2_ (50–270 ka) following Luethi *et al*.^56^. (**d**) I/Ca measured on *N. pachyderma* (s) tests from cores PS2547 and TC493. (**e**) Bulk sediment CaCO_3_ content from PS2547. Grey columns show the abundance of the benthic foraminifera *E. exigua* as number of tests per cm^3^ in core PS2547. Yellow shading highlights peak interglacial periods (including deglaciations), and blue shading marks glacial maxima and cooling intervals.

I/Ca values in the modern and late Holocene samples are lower than ∼2.5 μmol mol^−1^ at sites with O_2_ minima <70 μmol kg^−1^ in the upper ocean (0–500 m) (Fig. 2a). In contrast, recent planktonic foraminifera at sites with O_2_ minima >100 μmol kg^−1^ have I/Ca >4 μmol mol^−1^, regardless of species (Fig. 2a). At site TC493/PS2547, the *N. pachyderma* (s) I/Ca ratio is high (5–7 μmol mol^−1^) during the Holocene and MIS 5 relative to the lowest values (<2 μmol mol^−1^) during glacial MIS 2 and 6 (Fig. 3 and Supplementary Table 2).

## Discussion

A tremendous amount of work has been devoted to developing foraminiferal proxies for temperature and pH, using global calibrations derived from core-top samples (for example, the Mg/Ca seawater temperature proxy^41^). Low I/Ca ratios of planktonic foraminifera unambiguously reveal the presence of low-oxygen waters, but a global calibration approach cannot establish planktonic foraminifera I/Ca as a linearly quantitative proxy for the continuum of dissolved O_2_ concentration. Due to the stepwise nature of redox reactions^42^, quantitative IO_3_^−^ reduction does not occur before the dissolved oxygen is depleted to a certain threshold value, triggering nitrate reduction^43^. IO_3_^−^ concentrations at water depths matching planktonic foraminiferal habitats are often not in equilibrium with the *in situ* O_2_ concentrations, and O_2_ contents which are sufficiently low to initiate major IO_3_^−^ reduction may be detrimental to many species^44^. Instead, the I/Ca (recording the *in situ* IO_3_^−^ concentration) is determined by the depth habitat of the foraminifera and the upper ocean IO_3_^−^ mixing gradient. This mixing gradient is largely controlled by the surface water IO_3_^−^ concentration and the depth of the IO_3_^−^ reduction zone^28^. Nonetheless, a planktonic foraminifera proxy that semi-quantitatively approximates dissolved O_2_ concentrations, indicative of the presence of an OMZ, can still be highly valuable for the paleoceanography community.

Before interpreting the down-core record from site TC493/PS2547, we identify the characteristic I/Ca signals for modern OMZs. IO_3_^−^ depth profiles in the open ocean generally fall into two types (Fig. 2d): (1) the OMZ-type, with low surface water values and near-zero subsurface values in the OMZ; and (2) the normal open ocean type (for example, in a well-oxygenated water column), with relatively high surface water values and even higher subsurface values. A threshold O_2_ concentration will cause complete IO_3_^−^ reduction in the subsurface, and there may be a surface water IO_3_^−^ threshold concentration below which complete IO_3_^−^ reduction is likely to happen in the water column. Combined with modern water column IO_3_^−^ and O_2_ data, the I/Ca values measured on modern and late Holocene planktonic foraminifera consistently indicate that I/Ca <2.5 μmol mol^−1^ is equivalent to a surface water IO_3_^−^ concentration of <0.25 μmol l^−1^, thus providing a marker for the presence of oxygen-depleted water with a subsurface O_2_ concentration <20–70 μmol kg^−1^ (Fig. 2a–c).

Modern surface water IO_3_^−^ concentrations are influenced by productivity and the presence of a subsurface OMZ^25^,^28^. To visualize this relationship, we compiled surface water IO_3_^−^ concentrations from the literature and plotted them against the minimum O_2_ concentrations in the subsurface water (Fig. 2b). The IO_3_^−^ concentration broadly increases with the minimum O_2_ concentration when the surface water IO_3_^−^ concentration is >0.25 μmol l^−1^ (Fig. 2b). This correlation is likely a reflection of surface productivity versus subsurface respiration, because lower productivity leads to lower iodine uptake in surface water and less oxygen consumption by subsurface organic matter decomposition. In areas with a strong OMZ and near-zero O_2_ values, the surface water IO_3_^−^ concentrations are below 0.25 μmol l^−1^ (Fig. 2b). A partition coefficient *K*_d_ (*K*_d_=[I/Ca]/[IO_3_^−^] with units of [μmol mol^−1^]/[μmol l^−1^]) of ∼10 was reported from abiological calcite synthesis experiments^17^,^20^. Using this *K*_d_ value, an IO_3_^−^ concentration <∼0.25 μmol l^−1^ results in I/Ca values <∼2.5 μmol mol^−1^ in calcite. This estimate is consistent with modern I/Ca at OMZ Sites 658, 849 and 1242, as well as the last interglacial I/Ca value at Site 720 (Fig. 2a). Therefore, a surface water I/Ca value <2.5 μmol mol^−1^ indicates that a pronounced subsurface O_2_ minimum exerted the dominant control on the upper ocean IO_3_^−^ profile. This I/Ca threshold value does not seem to depend on foraminiferal species (Fig. 2a).

The O_2_ threshold for maintaining an OMZ-type IO_3_^−^ profile is useful for the paleoceanographic application of the planktonic I/Ca proxy. At O_2_ concentrations <20 μmol kg^−1^, microbial processes become dominant^29^, and IO_3_^−^ likely would be completely reduced to I^−^ since the reaction is biologically mediated^45^ (for example, ODP Sites 1242, 720 and 849 in Fig. 2a,c). ODP Site 658 is located at the northern edge of a shallow pocket of distinctively low-oxygen water with mean O_2_ concentrations of ∼70 μmol kg^−1^ in the upper 200 m (ref. 46), which may be sufficiently low to generate an OMZ-type iodate profile. Three species of planktonic foraminifera analysed at ODP Site 1242 show exceptionally low I/Ca ratios around 0.5 μmol mol^−1^, corresponding to an IO_3_^−^ concentration of ∼0.05 μmol l^−1^. Such a low IO_3_^−^ concentration is comparable to that reported for a location where an extreme hypoxic event occurred^47^. Moreover, this low IO_3_^−^ concentration implies that IO_3_^−^ reduction should occur shallower than at Site 849 and at two sites with classic OMZ-type IO_3_^−^ profiles (Eastern Equatorial Pacific^28^ and Arabian Sea^48^; Fig. 2c). A comparison of the O_2_ profiles of these sites reveals that the O_2_ threshold needs to be >50 μmol kg^−1^ to achieve a shallower IO_3_^−^ reduction at Site 1242. Therefore, we suggest that I/Ca values lower than ∼2.5 μmol mol^−1^ indicate O_2_ minima <20–70 μmol l^−1^. This O_2_ range cannot be further narrowed down with the available information, and we refer to this range as the O_2_ threshold for an OMZ-type IO_3_^−^ profile. However, the threshold behaviour of IO_3_^−^ reduction (relative to O_2_) in subsurface waters does not necessarily lead to step changes in down-core records of planktonic I/Ca. This is because planktonic foraminifera typically record the IO_3_^−^ mixing gradient in the top part of water column, above the O_2_-depleted zone where rapid step changes in IO_3_^−^ concentrations occur. Low planktonic I/Ca values may be driven by shoaling of O_2_-depleted water, and/or by increasing productivity, both of which could change gradually over time.

The available data from modern and late Holocene planktonic foraminifera suggest that the I/Ca ratio acts as a robust (paleo-) proxy for determining the signature of O_2_-depletion in the upper ocean (Fig. 2). At site TC493/PS2547, I/Ca was high (5–7 μmol mol^−1^) during the Holocene and interglacial MIS 5 when compared with the lowest values (<2 μmol mol^−1^) characterizing peak glacial periods MIS 2 and 6 (Fig. 3). Changes in salinity, temperature and foraminiferal habitat, most likely, are not the main drivers for this record (Supplementary Discussion). The glacial I/Ca values of *N. pachyderma* (s) are best explained by the presence of a water mass with a dissolved O_2_ content <70 μmol kg^−1^ close to, i.e., above or near, this site (Figs 2 and 3). We reiterate that the low I/Ca does not necessarily imply O_2_-depleted seawater within the foraminiferal habitat.

At present, CDW wells up to a water depth of approximately 250–300 m in the Amundsen Sea^31^ and has O_2_ concentrations notably lower than the top 200 m of the water column (Fig. 2c). Although the interpretation of absolute values of planktonic δ^13^C is far from straightforward in the seasonal ice zone (for example, disequilibrium from seawater^49^), it is reasonable to assume that CDW had a strong influence on the local water column during glacial periods, as its upwelling along the continental margin was probably responsible for the opening of the glacial polynyas. The CDW upwelling at site TC493/PS2547 today partly originates from Pacific Deep Water (PDW) moving southward from the equator, with a low-oxygen and high nutrient signature (Fig. 1)^50^,^51^. δ^30^Si data from fossil diatoms and sponges indicate higher silicic acid concentrations in the Pacific sector of the Southern Ocean during the LGM, which further imply that either the southward transport of PDW was more efficient or PDW was less ventilated than today^52^. So glacial CDW was likely more O_2_ depleted than during interglacials, and upwelling of this water contributed to the glacial I/Ca signal at site TC493/PS2547.

The oxidation of I^−^ to IO_3_^−^ is thought to take from a few months up to 40 years^53^. Long-distance transport of well-oxygenated deep water with low IO_3_^−^ concentrations (<0.25 μmol l^−1^) has not been documented in the modern ocean, but this scenario should be tested with further work on I^−^ oxidation kinetics. Today our site is bathed in CDW transported from a Pacific OMZ and the interglacial I/Ca values at site TC493/PS2547 do not show any remnant signal of the OMZ from the Pacific Ocean. On the basis of the knowledge about iodine speciation change in modern ocean, we interpret the observed glacial I/Ca values as a local signal, in principle, indicating the presence of a water mass with low O_2_ and low IO_3_^−^ vertically or horizontally close to the planktonic foraminiferal habitat.

**Figure 4:**
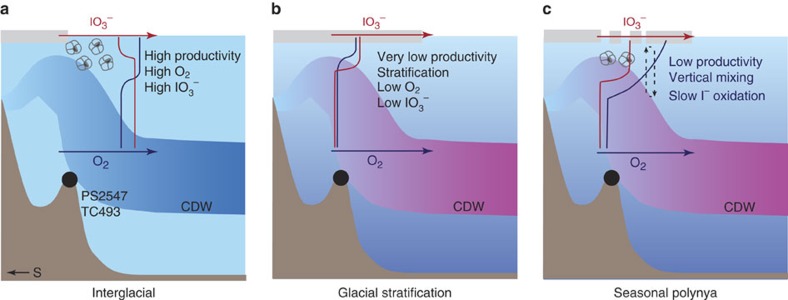
Conceptual illustration of paleo-environmental changes. Upper ocean IO_3_^−^ and O_2_ profiles were influenced by circulation, productivity and polynyas over glacial cycles. (**a**) Well-oxygenated interglacial condition; (**b**) Relatively oxygen-depleted glacial conditions with expanded sea–ice cover; (**c**) Episodic polynya opening during glacials.

In the setting of site TC493/PS2547 a coherent conceptual model for *N. pachyderma* (s) recording the presence/absence of O_2_-depletion needs to integrate changes in productivity, sea–ice extent and the opening/closing of polynyas on time scales of glacial to seasonal cycles (Fig. 4). Although the polynyas complicate the interpretation of the proxy data, their presence arguably provides the only window for sufficient accumulation of planktonic microfossils to record upper ocean conditions during glacial periods at such high latitudes.

The modern O_2_ profile at site TC493/PS2547 is defined by equilibration with the atmosphere at 0–250 m, and CDW influence below 250 m, as shown by the distinctively low O_2_ concentrations (Fig. 2c). With O_2_ above the threshold for complete IO_3_^−^ reduction in the entire water column, the IO_3_^−^ profile at site TC493/PS2547 should be similar to those at other high latitude locations, for example, site PS71/179–1 at 69°31′ S and 0°3′ W in the Weddell Sea^54^ (Fig. 2d). An interglacial scenario of relatively high seasonal productivity, high O_2_ and surface water IO_3_^−^ (>0.3 μmol l^−1^) concentrations (Fig. 4a), is well described for the modern Atlantic sector of Southern Ocean^54^.

Relative to the interglacial periods, the Southern Ocean experienced expanded sea–ice cover during glacial periods, and was less ventilated^9^,^36^. A more dynamic seasonal sea–ice cycle during ice ages would have increased water column stratification. Increased winter sea–ice formation (spatially and volumetrically) may have generated waters dense enough to sink ultimately to the bottom of the ocean^55^. On the other hand, melting of thicker sea ice during glacial-time summers in the seasonal sea–ice zone would have strengthened the halocline (not considering the influence of polynyas). So, the glacial seasonal stratification was likely stronger than today. These factors overall should have lowered the glacial O_2_ concentrations in the Southern Ocean (Fig. 4b). At site TC493/PS2547, glacial I/Ca demonstrate that the IO_3_^−^ profile was OMZ-like with complete IO_3_^−^ reduction near the foraminiferal habitat (Fig. 2d). However, the dynamics of polynyas must be considered when interpreting the location of the low O_2_ water mass, and the means by which the signal was recorded by *N. pachyderma* (s).

Without a polynya above site TC493/PS2547, glacial phytoplankton productivity under perennial sea–ice cover would have been relatively low due to the scarcity of light^34^, and planktonic foraminifera depending on algae could not flourish. The water column would have been relatively poorly ventilated and strongly stratified during these times, creating the ideal environment for developing low O_2_ conditions and an OMZ-type IO_3_^−^ profile (Fig. 4b). The episodic opening of a polynya re-established primary production (mainly by diatoms) and thus a planktonic foraminiferal habitat, vertical mixing and oxygenation in, at least, the uppermost part of the water column (Fig. 4c). While overall glacial-time production was reduced^30^,^34^, the planktonic foraminifera preserved in the glacial sediments probably recorded transient I/Ca changes in the water column associated with polynya-induced peaks in glacial productivity. Modern open ocean productivity pulses do not lower IO_3_^−^ concentrations to <0.25 μmol l^−1^ in oxygenated water (Supplementary Discussion)^54^, thus the glacial I/Ca signal is most likely driven by changes in O_2_ and not productivity.

The likely short-lived nature of glacial polynyas makes it difficult to envisage that very brief plankton blooms alone could produce a utilization-driven O_2_ depletion in a cold, well-oxygenated Southern Ocean. For the same reason, it is difficult to imagine that the vertical mixing cells restricted by the size of the polynya could rapidly oxygenate voluminous nearby waters outside of the polynya, if most of the sea–ice covered areas were O_2_-depleted. The more likely scenario is that the O_2_ concentrations in the deep and abyssal Southern Ocean were generally lower during glacial periods than during interglacial periods. Upwelling of a more O_2_-depleted CDW in the generally stratified upper ocean was mainly responsible for the IO_3_^−^ reduction at site TC493/PS2547 (Fig. 4b), while the episodic opening of polynyas created habitable conditions for planktonic foraminifera to record the deoxygenation in the upper ocean (Fig. 4c). We suggest that the I/Ca proxy should be used as a local proxy, in principle. However, it is probably a reasonable speculation that this record (Fig. 3) shows oxygenation changes integrated over a regional volume of water (e.g. CDW).

The timing of glacial deoxygenation and deglacial reoxygenation at site PS2547 shows potential linkages to global climate changes (Fig. 3). The appearance of OMZ-type I/Ca values (<∼2.5 μmol mol^−1^) during past glacial periods coincided with the lowering in atmospheric pCO_2_ level below the long-term mean value^56^. Identical timing was reported for a strongly stratified Antarctic Zone coincident with pCO_2_ decrease under the same threshold value (225 p.p.m.) in the Atlantic sector of the Southern Ocean^57^. Stronger stratification may be the common driving force for the productivity change (ODP Site 1094) and oxygenation change (PS2547/TC493) in the Antarctic zone. Furthermore, during the last interglacial period, the recovery of *N. pachyderma* (s) I/Ca values is offset from the δ^18^O trend, with peak I/Ca occurring about 10 kyr after the peak δ^18^O (Fig. 3), an observation worthy of future investigation.

Our I/Ca results build on other evidence^52^,^58^,^59^ to make a stronger case for lower oxygen concentrations in CDW (and very likely PDW) during glacial periods. Altogether with the reconstructed O_2_ content of deep waters in the glacial North Atlantic^10^, these observations seem to allude to large scale deoxygenation in the glacial global ocean interior^60^. Future work providing quantitative reconstructions of bottom water O_2_ concentrations in the Southern Ocean, especially south of the APF, and in other major ocean basins will shed new light on the mechanisms of sequestering atmospheric CO_2_ during ice ages.

## Methods

### Foraminifera cleaning

Sediments were sampled from the split core sections and wet sieved. Approximately 40 tests of *N. pachyderma* sinistral were picked from the 200–250 μm size fraction of each sample. The cleaning procedure followed the Mg/Ca protocol of Barker *et al*.^61^. Cleaned glass slides were used to gently crack open all chambers. Clay particles were removed in an ultrasonic water bath. After adding NaOH-buffered 1% H_2_O_2_ solutions the samples were heated in boiling water for 10–20 min to remove organic matter. Calcareous microfossils were then thoroughly rinsed with de-ionized water. Reductive cleaning was not applied because contribution of iodine from Mn-oxides is deemed negligible^19^.

### ICP-MS measurements

The cleaned samples were dissolved in 3% nitric acid, and diluted to solutions with 50 p.p.m. Ca for analyses. Iodine calibration standards were freshly prepared also with 50 p.p.m. Ca. 0.5% tertiary amine solution (Spectrasol, CFA-C) was added to stabilize iodine within a few minutes after the sample dissolution. The measurements were performed immediately after that to minimize potential iodine loss. The sensitivity of iodine was tuned to above 80 kcps for a 1 p.p.b. standard. The precision for ^127^I is typically better than 1%. The long-term accuracy is guaranteed by frequently repeated analyses of the reference material JCp-1 (ref. 17). The detection limit of I/Ca is on the order of 0.1 μmol mol^−1^. The I/Ca measurements were performed using a quadrupole ICP-MS (Bruker M90) at Syracuse University.

## Additional information

**How to cite this article:** Lu, Z. *et al*. Oxygen depletion recorded in upper waters of the glacial Southern Ocean. *Nat. Commun.* 7:11146 doi: 10.1038/ncomms11146 (2016).

## Supplementary Material

Supplementary Information Supplementary Figures 1-2, Supplementary Tables 1-2, Supplementary Discussion and Supplementary References.
