# Erratum: Oxygen depletion recorded in upper waters of the glacial Southern Ocean

**DOI:** 10.1038/ncomms11531

**Published:** 2016-04-26

**Authors:** Zunli Lu, Babette A. A. Hoogakker, Claus-Dieter Hillenbrand, Xiaoli Zhou, Ellen Thomas, Kristina M. Gutchness, Wanyi Lu, Luke Jones, Rosalind E. M. Rickaby

Nature Communications 7: Article number: 11146 10.1038/ncomms11146 (2016); Published: 03312015; Updated: 04262015

In Fig. 3 of this Article, data in panel ‘a' were inadvertently partially obscured during the production process. The correct version of Fig. 3 now appears in the PDF and HTML versions of the Article.

