# Supplementary material for: Oxygen depletion recorded in upper waters of the glacial Southern Ocean
Source: Nat Commun. 2016 Mar 31;7:11146. doi: 10.1038/ncomms11146 (PMC4821880; doi:10.1038/ncomms11146)
Supplement: Supplementary Information — Supplementary Figures 1-2, Supplementary Tables 1-2, Supplementary Discussion and Supplementary References. [file ncomms11146-s1.pdf]

Supplementary Information

Supplementary Figures

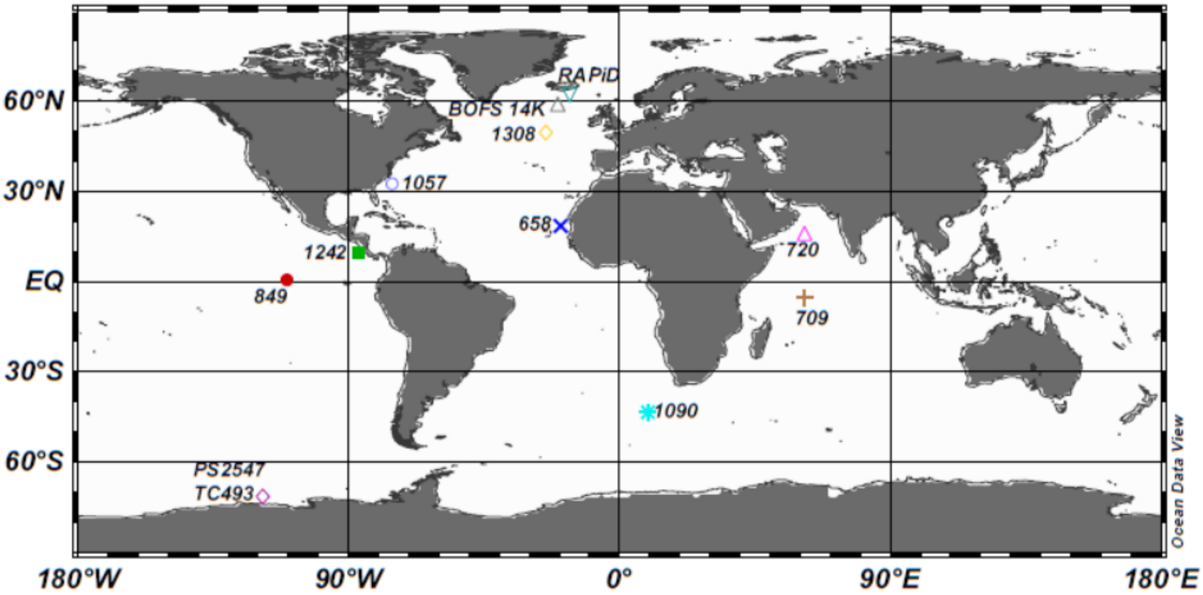

Supplementary Figure 1. Site map for core-top, Late Holocene and MIS 5 samples.

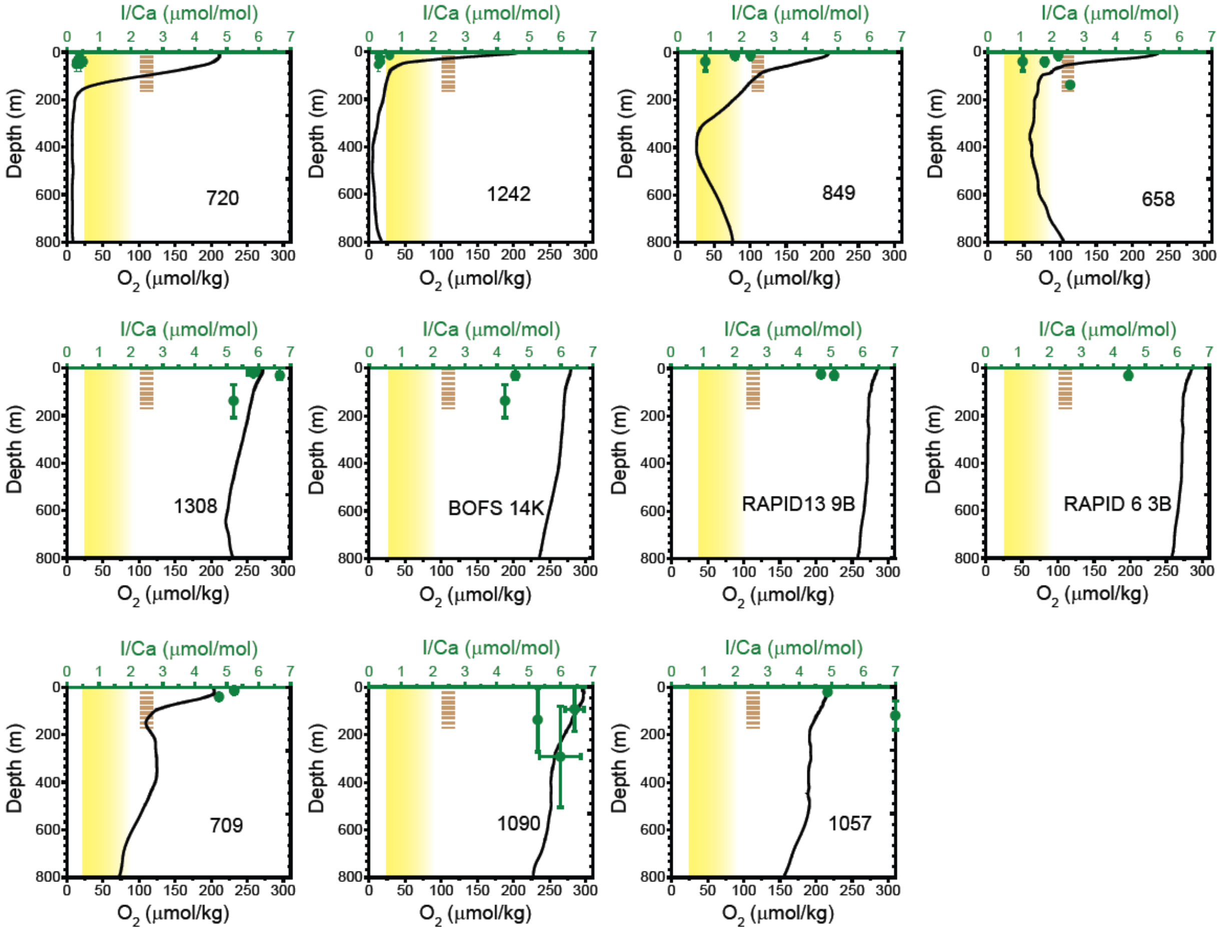

Supplementary Figure 2. I/Ca in core-top and Late Holocene samples compared with water column O_2 (Note: I/Ca data at Site 720 are from MIS 5 samples). Brown dashed line marks 2.5 μmol/mol and yellow box indicates O_2 threshold for iodate reduction.

Supplementary Tables

Supplementary Table 1. Core top and Late Holocene planktonic I/Ca data.

Species	**Site/Sample**	**Depth (cm)**	**I/Ca**	**±**
G. bulloides	ODP 1090A 1H 1W	0-3	6.43	0.30
G. inflata	ODP 1090A 1H 1W	0-3	5.28	
G. truncatulinoides	ODP 1090A 1H 1W	0-3	5.99	0.64*
G. ruber	ODP 658	0-8	2.21	
G. inflata	ODP 658	0-8	2.58	
G. menardii	ODP 658	0-8	1.78	
N. dutertrei	ODP 658	0-8	1.09	
G. sacculifer	709A1H1W	0-1	5.24	
P. obliquiloculata	709A1H1W	0-1	5.24	
G. menardii	709A1H1W	0-1	4.76	
N. dutertrei	849A1H1W16	16-18	0.86	
P. obliquiloculata	849A1H1W16	16-18	2.27	
G. sacculifer	849A1H1W16	16-18	1.79	
G. bulloides	ODP 1308C 1H 1A	0-3	6.67	
G. inflata	ODP 1308C 1H 1A	0-3	5.22	
O. universa	ODP 1308C 1H 1A	0-3	5.84	0.18
G. sacculifer	1242A1H1A 32-34	32-34	0.64	
O. universa	1242A1H1A 32-34	32-34	0.30	
G. menardii	1242A1H1A 32-34	32-34	0.36	
N. dutertrei	1242A1H1A 32-34	32-34	0.29	
G. bulloides	BOFS 14K	0-1	4.58	
G. inflata	BOFS 14K	0-1	4.25	
G. bulloides	RAPID 6 3B	0-1	4.47	
N. pachyderma (dex)	RAPiD 13 9B	0-1	4.70	
G. bulloides	RAPiD 13 9B	0-1	5.11	
G. menardii	720A 1H 1W	20-22	0.40	0.1
N. dutertrei	720A 1H 1W	20-22	0.35	0.1
G. sacculifer	720A 1H 1W	20-22	0.39	
O. universa	ODP 1057A 1 H 1A	0-2	4.88	
G. tumida	ODP 1057A 1 H 1A	0-2	7.00	

* error for *G. truncatulinoides* at ODP Site 1090 was calculated from measurements on two different size fractions (250-300 μm vs. >300 μm).

Supplementary Table 2. PS2547 and TC493 data plotted in Fig. 3. Age fix points are bolded.

					PS2547					
**Depth**	**Age**	**I/Ca**	**±**	**CaCO3**	***N. pachyderma*** **d18O**	***N. pachyderma*** **d13C**	***C. cf wuellerstorfi*** **d18O**	***C. cf. wuellerstorfi*** **d13C**	***E. exigua*** **abundance**
**m**	**kyr**	**μmol /mol**	**μmol /mol**	**%**	**‰**	**‰**	**‰**	**‰**	**per cm^3**
0.02	11.3			15.7	4.36	0.96	3.4	0.86	
0.05	13.3	2.3		5.9	4.51	0.8	4.13	0.21	
0.07	14.7	1.5		2	4.75	0.63	4.27	0.36	
0.09	16.0	1.7		1.3	4.68	0.35	4.32	0.29	
0.12	**18.0**			1.1	5.1	0.33	4.46	0.27	
0.16	31.9	2.5		11.8	5.01	0.33	4.02	0.25	
0.21	**45.0**	2.6		21	4.69	0.41	3.77	0.37	16
0.25	**60.0**	2.6		38.6	4.73	0.5	4.09	0.33	1
0.29	82.9	3.9		45.4	4.27	0.34	3.72	0.48	8
0.32	100.1	5.0		37.6	4.42	0.53	3.98	0.38	5
0.36	**123.0**	4.4		29.3	4.02	0.54	3.5	0.44	4
0.39	125.6	2.9		11.1	4.18	0.78	3.5	0.63	9
0.44	129.9	2.5		4.6	4.24	0.74	3.86	0.25	2
0.47	132.4			2.8	4.56	0.35	4.38	0.07	
0.5	**135.0**	1.7		5.6	4.9	0.22	4.44	0.07	9
0.55	143.3	2.3		5	4.71	0.04	4.24	0.02	
0.6	151.7	1.8		4.2	4.95	0.16	4.24	-0.03	5
0.65	160.0	2.5		3.9	4.56	0.04	4.22	0.02	
0.7	168.3	2.3	0.3	5.7	4.76	0.13	4.32	-0.1	
0.75	176.7	4.0		20.9	4.54	0.2	4.1	-0.14	
0.8	**185.0**	3.2		31.3	4.73	0.1	4.66	0.32	
0.85	191.6	4.4		35.6	4.39	0.05	3.75	0.12	20
0.9	**199.0**	4.3	0.2	34.9	4.31	0.55	3.74	0.54	9
0.95	**215.0**	3.7		35.9	4.3	0.16	3.62	0.27	11
1	227.0	2.4		30.2	4.5	0.45	3.85	0.13	13
1.05	239.0	2.8		22.8	4.32	0.34	3.85	0.17	22

TC493

Depth	Age	I/Ca	±	*N. pachyderma* d18O	*N. pachyderma* d13C	*C. cf wuellerstorfi* d18O	*C. cf. wuellerstorfi* d13C
m	kyr	μmol/mol	μmol/mol	‰	‰	‰	‰
0.005	8.3	5.31		3.62	1.20	3.44	0.84
0.015	8.9	5.35	0.11	3.93	0.82	3.56	0.81
0.025	9.5	4.01		3.84	1.28	3.64	0.47
0.035	10.1	2.48		3.93	0.94	4.09	0.42
0.045	10.7	3.26		4.07	0.78	3.79	0.55
0.055	11.3	2.58		4.41	0.81	4.16	0.25
0.065	11.9	2.09		4.41	0.71	4.37	0.48
0.075	12.5	2.20		4.46	0.74	4.38	0.29
0.085	13.2	2.03		4.62	0.54	4.57	0.39
0.095	13.8	1.73		4.50	0.68	4.32	0.44
0.105	14.4	1.42		4.59	0.58	4.53	0.31
0.115	15.0	1.21		4.77	0.41	4.39	0.43
0.125	15.6	1.12		4.91	0.38	4.45	0.44
0.135	16.2	1.67		4.82	0.56	4.49	0.23
0.145	16.8	1.96		4.63	0.35	4.27	0.37
0.165	**18.0**	1.40		5.02	0.35	4.51	0.38
0.175	19.8	1.33					
0.185	21.5	1.84		4.84	0.52	4.70	0.11
0.195	23.3	1.71		4.96	0.41	4.43	0.38
0.205	25.0	1.55					
0.235	30.3			4.71	0.40	4.38	0.66
0.315	44.4	2.51		5.04	0.32	4.63	0.20
0.325	46.2	2.43		5.10	0.22	4.42	0.31
0.335	48.0	2.41		5.00	0.25	4.58	0.24
0.345	49.7	3.01		5.01	0.40	4.63	0.20
0.355	51.5	3.46		5.01	0.17	4.66	0.22
0.365	53.2	2.85		5.10	0.25	4.50	0.36
0.375	**55.0**	2.65		4.69	0.27	4.54	0.21
0.385	57.0	2.53		4.78	0.48	4.53	0.33
0.395	58.9	3.11		4.87	0.38	4.30	0.33
0.405	60.9	3.20		4.81	0.45	4.31	0.14
0.415	62.8	2.75		4.90	0.37	4.48	0.28
0.425	64.8	3.18		4.82	0.43	4.38	0.28
0.435	66.7	3.31		4.70	0.36	4.29	0.38
0.445	68.7	3.29		4.59	0.42	4.31	0.32
0.455	70.6	3.28		4.67	0.30	4.39	0.24
0.465	72.6	3.29		4.67	0.38	4.28	0.48
0.475	74.5	3.76		4.85	0.41	4.34	0.42

0.485	76.5	4.18	4.40	0.31	4.33	0.30
0.495	78.5	3.51	4.69	0.50	4.17	0.41
0.505	80.4	3.21	4.55	0.41	4.03	0.42
0.515	82.4	3.39	4.63	0.49	4.15	0.37
0.525	84.3	3.03	4.62	0.47	4.00	0.46
0.535	86.3	2.81	4.75	0.44	4.36	0.37
0.545	88.2	3.16	4.54	0.45	4.08	0.33
0.555	90.2	3.88	4.42	0.50	4.18	0.22
0.565	92.1	3.80	4.66	0.48	4.18	0.42
0.575	94.1	4.48	4.49	0.42	4.00	0.28
0.585	96.0	4.94	4.37	0.50	3.86	0.31
0.595	**98.0**	5.17	4.18	0.71	3.94	0.55
0.605	101.7	4.95	4.28	0.54	3.64	0.89
0.615	105.3	6.07	4.25	0.68	3.79	0.53
0.625	**109.0**	6.26	4.39	0.65	3.84	0.45
0.635	111.3	5.48	4.12	0.66	3.54	0.43
0.645	113.7	6.60	4.28	0.52	3.74	0.45
0.655	116.0	6.67	4.07	0.66	3.40	0.57
0.665	118.3	6.01	4.01	0.58	3.23	0.37
0.675	120.7	5.36	3.91	0.79	3.11	0.55
0.685	**123.0**	4.73	3.78	0.80	3.39	0.34
0.695	123.9	3.11	4.23	0.70	3.44	0.52
0.705	124.9	4.04	4.40	0.53	3.16	0.50
0.715	125.8	4.81	4.10	0.54	3.23	0.54
0.725	126.8	3.79	4.50	0.40	3.95	0.22
0.735	127.7	4.37	4.22	0.64	3.86	0.45
0.745	128.6	2.64	4.44	0.56	4.03	0.40
0.755	129.6	2.15	4.80	0.38	4.24	0.27
0.765	130.5	2.28	4.80	0.30	4.42	0.11
0.775	131.4	3.93	4.63	0.34	4.09	0.42
0.785	132.4	3.54	4.83	0.35	4.50	0.09
0.795	133.3	3.91	4.37	0.25	4.35	0.10
0.805	134.3	3.22	4.65	0.25	4.62	0.15
0.815	135.2	3.53	4.71	0.15	4.57	0.13
0.825	136.1	2.80	5.00	0.25	4.45	0.14
0.835	137.1	3.41	5.12	0.06	4.45	0.09
0.845	**138.0**	2.20	5.16	0.04	4.48	0.07

Supplementary Discussions

The iodine (IO_3^-) concentration in open ocean seawater can be influenced by freshwater input or brine formation which are shown in salinity changes. In the study area, the modern seawater salinity in the upper 300 m ranges between 33.5 and 34.5 PSU. Whole ocean salinity changes over glacial cycles [1] could only have caused small variations in IO_3^- concentrations and thus I/Ca changes that are much less than those recorded in the planktic foraminifera from site TC493/PS2457. Also salinity during glacials should slightly increase the I/Ca ratio and therefore cannot explain the observed large decrease.

The uptake of trace element into foraminifera tests is often temperature dependent (for example, U/Ca[2]). The K_d for IO_3^- incorporation in synthetic calcite seems to increase with a decrease in temperature (Zhou et al., 2014 supplementary information [3]). If this observation is applicable to foraminifera tests, then the glacial I/Ca values at site TC493/PS2547 would have been even lower without the temperature effect. The I/Ca values of core-top planktonic foraminifera from the well-oxygenated high latitude N. Atlantic are similar to the I/Ca values of core-top planktonic foraminfera at Site 709 in the low latitude Indian Ocean, but the upper ocean temperature difference could be greater than 15°C depending on the foraminifera habitats. These observations suggest that temperature has no dominant influence on foraminifera I/Ca, although this hypothesis needs to be confirmed by more core top data or culture experiments.

N. pachyderma sin. can survive winter seasons in sea ice [4,5], leading to a potential concern of glacial I/Ca signatures associated with a planktonic foraminifera habitat in brine channels within sea ice instead of the underlying water column. However, there is no evidence that *N. pachyderma* sin. can actually calcify or reproduce within the sea ice [6]. Moreover, the presence of epibenthic foraminifera with $\delta^{18}O$ values typical for glacial periods in the sediments of TC493/PS2547 documents that a significant flux of food particles to the seafloor must have occurred at least episodically during these times in response to phytoplankton production, likely within polynyas [7]. The living habitat of *N. pachyderma* sin. at this site is probably 0-200 m [8], recording sub-surface water conditions.

While overall glacial-time production was reduced in the Amundsen Sea [7,9,10], the planktonic foraminifera preserved in the glacial sediments at site TC493/PS2547 probably recorded transient I/Ca changes in the water column associated with polynya-induced peaks in glacial productivity. If the low glacial I/Ca values were snapshots of water column conditions, they may be related to iodine uptake under high productivity or OMZ conditions. To our knowledge, there are no observations from the modern open ocean that productivity pulses could lower IO_3^- concentrations to <0.25 μmol/l. Bluhm et al. [11] collected seawater samples in the Weddell Sea one month after an intense phytoplankton bloom and found IO_3^- concentrations mostly >0.4 μmol/l. Thus, open ocean plankton blooms are unlikely to reduce surface water IO_3^- concentrations to <0.25 μmol/l. If the proposed glacial productivity pulse at site TC493/PS2547 decreased surface water IO_3^- concentration significantly (as recorded by the low I/Ca ratios of the foraminifera), then the transient organic matter flux and associated O_2 utilization would also have contributed to the formation of a short-lived OMZ.

Supplementary References:

1 Adkins, J. F., McIntyre, K. & Schrag, D. P. The salinity, temperature, and delta O-18 of the glacial deep ocean. *Science* **298**, 1769-1773 (2002).

2 Yu, J. M., Elderfield, H., Jin, Z. D. & Booth, L. A strong temperature effect on U/Ca in planktonic foraminiferal carbonates. *Geochimica et Cosmochimica Acta* **72**, 4988-5000, doi:DOI 10.1016/j.gca.2008.07.011 (2008).
3 Zhou, X. L., Thomas, E., Rickaby, R. E. M., Winguth, A. M. E. & Lu, Z. L. I/Ca evidence for upper ocean deoxygenation during the PETM. *Paleoceanography* **29**, 964-975, doi:Doi 10.1002/2014pa002702 (2014).
4 Spindler, M. & Dieckmann, G. S. DISTRIBUTION AND ABUNDANCE OF THE PLANKTIC FORAMINIFER NEOGLOBOQUADRINA-PACHYDERMA IN SEA ICE OF THE WEDDELL SEA (ANTARCTICA). *Polar Biology* **5**, 185-191, doi:10.1007/bf00441699 (1986).
5 Dieckmann, G. S., Spindler, M., Lange, M. A., Ackley, S. F. & Eicken, H. ANTARCTIC SEA ICE - A HABITAT FOR THE FORAMINIFER NEOGLOBOQUADRINA-PACHYDERMA. *Journal of Foraminiferal Research* **21**, 182-189 (1991).
6 Berberich, D. Die planktische Foraminifere Neogloboquadrina pachyderma (Ehrenberg) im Weddellmeer, Antarktis. Berichte zur Polarforschung - Reports on Polar Research, 195, Alfred-Wegener-Institut, Bremerhaven. (1996).
7 Thatje, S., Hillenbrand, C. D., Mackensen, A. & Larter, R. Life hung by a thread: Endurance of antarctic fauna in glacial periods. *Ecology* **89**, 682-692, doi:10.1890/07-0498.1 (2008).
8 Mortyn, P. G. & Charles, C. D. Planktonic foraminiferal depth habitat and delta O-18 calibrations: Plankton tow results from the Atlantic sector of the Southern Ocean. *Paleoceanography* **18**, doi:10.1029/2001pa000637 (2003).
9 Hillenbrand, C. D., Futterer, D. K., Grobe, H. & Frederichs, T. No evidence for a Pleistocene collapse of the West Antarctic Ice Sheet from continental margin sediments recovered in the Amundsen Sea. *Geo-Marine Letters* **22**, 51-59, doi:10.1007/s00367-002-0097-7 (2002).
10 Hillenbrand, C. D., Kuhn, G. & Frederichs, T. Record of a Mid-Pleistocene depositional anomaly in West Antarctic continental margin sediments: an indicator for ice-sheet collapse? *Quaternary Science Reviews* **28**, 1147-1159, doi:10.1016/j.quascirev.2008.12.010 (2009).
11 Bluhm, K., Croot, P. L., Huhn, O., Rohardt, G. & Lochte, K. Distribution of iodide and iodate in the Atlantic sector of the southern ocean during austral summer. *Deep-Sea Research Part Ii-Topical Studies in Oceanography* **58**, 2733-2748, doi:10.1016/j.dsr2.2011.02.002 (2011).
